# The Role of Cerebrovascular-Reactivity Mapping in Functional MRI: Calibrated fMRI and Resting-State fMRI

**DOI:** 10.3389/fphys.2021.657362

**Published:** 2021-03-25

**Authors:** J. Jean Chen, Claudine J. Gauthier

**Affiliations:** ^1^Baycrest Centre for Geriatric Care, Rotman Research Institute, Toronto, ON, Canada; ^2^Department of Medical Biophysics, University of Toronto, Toronto, ON, Canada; ^3^Department of Physics, Concordia University, Montreal, QC, Canada; ^4^Montreal Heart Institute, Montreal, QC, Canada

**Keywords:** calibrated BOLD, neurovascular coupling, cerebrovascular reactivity, resting-state fMRI, functional connectivity

## Abstract

Task and resting-state functional MRI (fMRI) is primarily based on the same blood-oxygenation level-dependent (BOLD) phenomenon that MRI-based cerebrovascular reactivity (CVR) mapping has most commonly relied upon. This technique is finding an ever-increasing role in neuroscience and clinical research as well as treatment planning. The estimation of CVR has unique applications in and associations with fMRI. In particular, CVR estimation is part of a family of techniques called calibrated BOLD fMRI, the purpose of which is to allow the mapping of cerebral oxidative metabolism (CMRO2) using a combination of BOLD and cerebral-blood flow (CBF) measurements. Moreover, CVR has recently been shown to be a major source of vascular bias in computing resting-state functional connectivity, in much the same way that it is used to neutralize the vascular contribution in calibrated fMRI. Furthermore, due to the obvious challenges in estimating CVR using gas challenges, a rapidly growing field of study is the estimation of CVR without any form of challenge, including the use of resting-state fMRI for that purpose. This review addresses all of these aspects in which CVR interacts with fMRI and the role of CVR in calibrated fMRI, provides an overview of the physiological biases and assumptions underlying hypercapnia-based CVR and calibrated fMRI, and provides a view into the future of non-invasive CVR measurement.

## Bold Signal Physiology

Functional MRI (fMRI) is predominantly performed using the blood-oxygenation level-dependent (BOLD) signal. This signal is based on the paramagnetic properties of deoxyhemoglobin, providing a sensitive, but un-specific marker of neuronal activity. This lack of specificity stems from the fact that most deoxyhemoglobin (dHb) locally arises from baseline metabolism, with a more modest contribution from task-evoked neuronal activity. The signal measured during a task is due to the dilution of these two sources of dHb from a feedforward cascade of events leading to vasodilation in arterioles, bringing fully oxygenated, and therefore diamagnetic, blood to the area of activity (Girouard and Iadecola, 2006; Iadecola, 2017). Therefore, rather than being a direct marker of neuronal activity, the BOLD signal reflects the relative interplay between baseline oxidative metabolism, task-evoked metabolism, neurovascular coupling mechanisms, and the extent to which local vessels dilate in response to these neurovascular coupling chemical signals (Gauthier and Fan, 2019). It is this last aspect that underlies the amplitude of the cerebrovascular reactivity (CVR) response measured by BOLD fMRI.

While BOLD-based fMRI is widely used and has several applications in clinical fields (Chen, 2018, 2019; Gauthier and Fan, 2019; Specht, 2019), its physiologically unspecific nature makes it vulnerable to a variety of biases, especially in clinical populations (Ances et al., 2011; De Vis et al., 2015; Lajoie et al., 2017; Mazerolle et al., 2018; Chen, 2019). These biases can be vascular in nature, such as differences in baseline blood flow or reactivity, which are known to be prevalent in aging and clinical populations, or maybe due to decline in the availability of neuronal resources. It has been estimated that in healthy brains, the vascular response component is about twice the amplitude of the metabolic response (Hoge et al., 1999a; Uludağ et al., 2004). The vascular response consists of both a blood flow and a blood volume response, but because blood flow has a supralinear dependence on vessel diameter (modeled using Poiseuille’s law), it is the blood flow response that dominates the BOLD response. In less healthy populations, changes in vascular elasticity or neurovascular coupling mechanisms can lead to reduced vasodilation (Girouard and Iadecola, 2006; Iadecola, 2017). Because of this supralinear dependence on diameter, even small differences in diameter changes with aging or disease can have a large impact on blood flow as compared to young healthy populations. Therefore, the different physiological sub-components that make up the BOLD signal may not contribute identically to the measured signal in populations of different ages or presenting with different health conditions. This can lead to systematic biases in many BOLD signal comparisons across groups (Gauthier et al., 2012).

### BOLD Sensitivity to CO_2_

In the past decades, the CO_2_-driven BOLD response has been the preeminent method for mapping CVR. CO_2_ is a potent vasodilator used that has been shown to rely mainly on the nitric oxide (NO) pathway to increase arterial diameter (Pelligrino et al., 1999; Najarian et al., 2000; Peebles et al., 2008; Iadecola, 2017). While the exact source of NO (endothelial, neuronal or astrocytic) is still debated, NO production has been shown to mirror changes in CO_2_ partial pressure, with for example a 40% increases in CO_2_ partial pressure resulting in a 36% increase in NO production through endothelial cells in Fathi et al. (2011). Vessel diameter is highly sensitive to the surrounding CO_2_ concentration, with increasing CO_2_ partial pressures leading to linear increases in both vessel diameter and flow (Hülsmann and Dubelaar, 1988; Komori et al., 2007). In Komori et al. (2007) for example, this increase was shown to be of 21.6% for arteriolar diameter and 34.5% flow velocity for a 50% change in CO_2_ partial pressure in rabbit arterioles. This sensitivity can be captured using MRI, within our own data, a 12.0% change in inhaled CO_2_ concentration resulting in a 24.9% change in gray matter CBF measured using arterial spin labeling (ASL) and a 1.5% change in the gray matter BOLD signal (Gauthier and Hoge, 2013). Since the BOLD signal has both a static and temporal signal-to-noise ratio (SNR) that is typically above 100 (Triantafyllou et al., 2005; Gauthier and Hoge, 2013), it is a sensitive measure of CO_2_-induced vasodilation at the whole-brain level.

To induce a BOLD response to CO_2_, either hypercapnia and hypocapnia could be used. Hypercapnia is easier to achieve with more robust BOLD responses, while hypocapnia can be achieved with prospective targeting (Halani et al., 2015), hyperventilation (Cohen et al., 2002), or cued deep breathing (Bright et al., 2009). The various methods for producing CO_2_ variations are summarized in a number of recent reviews (Fierstra et al., 2013; Chen, 2018; Pinto et al., 2020).

## The Role of CVR in Calibrated fMRI

There exists a variety of methods to extract or correct the BOLD signal and make it a more quantitative marker of neuronal activity (Hoge, 2012; Gauthier and Fan, 2019). Notably, CVR is a key component of a family of techniques called calibrated fMRI, which is the predominant approach to quantify and extract the neuronal and vascular components of the BOLD response (Davis et al., 1998; Hoge et al., 1999a, b; Chiarelli et al., 2007; Gauthier and Hoge, 2012). In its fuller implementations, calibrated fMRI allows the separation of the BOLD signal into its baseline and task-induced vascular and metabolic components (Bulte et al., 2012; Gauthier and Hoge, 2012; Wise et al., 2013). In this review, the role of CVR in calibrated fMRI will be discussed.

### What Is Calibrated fMRI?

In its most common form, calibrated fMRI uses breathing manipulations to estimate the blood flow and blood volume component of the BOLD response to a task, in order to separate it from the non-vascular component of the BOLD signal (Davis et al., 1998; Hoge et al., 1999a, b; Chiarelli et al., 2007; Gauthier and Hoge, 2012). The most common calibration procedure for this type of technique uses hypercapnia or increased CO_2_ concentration in inhaled air, to cause a putatively purely vascular response (Davis et al., 1998; Hoge et al., 1999a). This calibration procedure is based on the underlying assumption that CO_2_, known to be a potent vasodilator, does not cause any change in oxidative metabolism. This vascular CO_2_ response is essentially CVR.

(1) $$M=\frac{\Delta \,B\,O\,L\,D/B\,O\,L\,D_{0}}{1-{\left(C\,B\,F/C\,B\,F_{0}\right)}^{\alpha -\beta }}$$

The original calibrated fMRI model was presented by Davis et al. (1998), followed in 1999 by a more complete description of the dHb dilution model that underlies it by Hoge et al. (1999a). In this model, the BOLD signal measured during hypercapnia is related to the CBF signal measured using ASL during hypercapnia, the calibration M parameter, and two other parameters typically assumed from the literature: α, which represents flow-volume coupling, and β, which represents the field strength-dependent magnetic properties of dHb. The M parameter is given by Eq. 1. The BOLD and CBF components can be measured, while alpha and beta are assumed, and M is the output of this calibration procedure. Conceptually, M represents the maximum possible BOLD signal. Since hypercapnia is assumed to be metabolically neutral, then M corresponds to the BOLD signal one would obtain if all dHb present in the brain from baseline metabolism were suddenly removed. At 3T, this value has been found to be between 4 and 12% when using a hypercapnia model see review in Hoge (2012), Blockley et al. (2015), Mark et al. (2015), and Gauthier and Fan (2019). To perform a calibrated fMRI experiment, therefore, one measures the BOLD and CBF percent signal change in response to mild hypercapnia, then uses the calibration equation from the model to extrapolate to the asymptote of the curve, corresponding to this maximal dilution of dHb. CVR is an intermediate measurement of this technique since it is measured as the BOLD or CBF percent change per mmHg change in CO_2_ concentration during hypercapnia.

The next step of the calibrated fMRI framework per the Davis model is then to estimate the oxidative metabolism component of the BOLD signal measured in response to a task, by combining the M parameter already measured, the BOLD signal measured in response to the task, the CBF signal measured in response to the same task, and the same alpha and beta parameters mentioned in the calibration procedure. These alpha values are assumed to be the same for the task and calibration (essentially CVR) procedures in most cases, though some work has shown that these may in fact be different (Chen and Pike, 2009, 2010b). It is also noteworthy that some work has suggested that the model should be treated as a heuristic model, rather than a biophysical model and that the value of alpha and beta can in fact be determined through data fitting, resulting in a different set of values than what has typically been used in the literature (Griffeth and Buxton, 2011).

Other versions of calibrated fMRI have been developed following this initial formulation. The simplest version consists in normalizing the BOLD signal from a task by the measured CVR (Bandettini and Wong, 1997; Biswal et al., 2007; Liu et al., 2013). These other models are based on other breathing manipulations such as hyperoxia (Chiarelli et al., 2007) or a combination of hypercapnia and hyperoxia (Gauthier and Hoge, 2012). While hyperoxia-based calibration improves comfort and has been shown to lead to reliable estimates of M and CMRO_2_, there is evidence that this model underestimates the true M and CMRO_2_ (Gauthier and Hoge, 2012). Furthermore, this implementation does not allow estimation of CVR as an intermediate byproduct, which may be valuable in several clinical populations. Finally, extensions of these calibrated fMRI models have also been developed to measure metabolism at rest (Bulte et al., 2012; Gauthier and Hoge, 2012; Wise et al., 2013). These other techniques have been reviewed elsewhere (Gauthier and Fan, 2019).

### The Role of CVR in Calibrated fMRI

The most common calibrated fMRI technique is based on the important assumption that CO_2_ inhalation is metabolically neutral. At high doses, CO_2_ is likely to cause changes in metabolism, but it is typically assumed that the smaller concentrations used in calibrated fMRI (on the order of 5% CO_2_ in most cases) cause negligible changes in metabolism. This is a debated assumption, however, with some studies showing decreased metabolism during CO_2_ inhalation (Xu et al., 2011; Driver et al., 2017) and some showing no change (Chen and Pike, 2010a; Jain et al., 2011). Aside from the difficulty in proving the null hypothesis that CO_2_ is metabolically neutral, whether metabolic activity is detected in response to CO_2_ may be dependent on the technique and the hypercapnia level used to measure it. If hypercapnia does impact oxidative metabolism, and thus biases the CVR estimate used in the calibration step, an accurate measurement of this bias is crucial, as it has been shown to have a large impact on the output of the calibrated fMRI model (Blockley et al., 2015). However, correction of the model to account for the change in CMRO_2_ would be straightforward should an accurate measurement of this effect arise as a CMRO_2_ change parameter could be added to the M equation rather than assuming a value of one (Blockley et al., 2015; Driver et al., 2017).

Another important consideration is the presence of non-linearities and spatial heterogeneities in the BOLD and underlying CBF response to hypercapnia. It has been shown that the dose-response curve for the CBF and BOLD responses to graded hypercapnia follows a sigmoidal shape, with lower CBF and BOLD responses in the hypocapnic range, as well as saturation effects in the very high inhaled CO_2_ concentration range (Tancredi et al., 2012; Duffin et al., 2017). Therefore, correction by CVR or calibrated fMRI must be interpreted with caution when operating outside the more linear range around 35–50 mmHg (Tancredi et al., 2012). Furthermore, the BOLD, and perhaps even more so, the CBF response to hypercapnia has been shown to be spatially heterogeneous (Gauthier et al., 2012; Tancredi et al., 2012; De Vis et al., 2015). Because of this, it is crucial that regional or voxel-wise measurements of CVR or M be used to validly inform the BOLD signal or performing calibrated fMRI.

### Assumptions in Calibrated fMRI

The Davis model also assumes that arterial blood is fully oxygenated. This is generally a reasonable assumption, as normal oxygen saturation for arterial blood is typically in the range of 97–100% in young subjects (Barratt-Boyes and Wood, 1957). However, this assumption may be problematic in older (Hardie et al., 2004) and diseased populations (Cukic, 2014; Slowik and Collen, 2020), which could suffer from lower saturations. However, modeling of the effects of anemia has shown that anemia has a very limited impact on the results of the model (Blockley et al., 2015).

Another underlying assumption of this technique is that the chemical signaling that underlies the neurovascular response is comparable to the signaling that underlies CO_2_-mediated vasodilation. This is because unless these two types of signaling are comparable, then using hypercapnia to assess the vascular response corresponding to a functional task may be misleading and be associated with the very systematic biases between populations that calibrated fMRI was designed to address. Neurovascular coupling is a complex orchestration of signals with many cell types and pathways involved. Detailed studies have shown that when they are active, neurons and interneurons release NO, and that this leads to vasodilation at the arteriolar level (Faraci and Brian, 1994; Rancillac et al., 2006; Attwell et al., 2010; Iadecola, 2017). Capillary dilation is, however, dependent on astrocytic activation of other pathways, especially the arachidonic acid pathway (Mishra et al., 2016). Nevertheless, it has been shown that inhibition of nNOS leads to an almost complete diminution of the BOLD and CBF response to neuronal stimulation in rat (Stefanovic et al., 2007), establishing nNOS as one of the main mediators of the neurovascular coupling that underlies the fMRI signals.

The vasodilatory response to hypercapnia on the other hand has been shown to be predominantly due to activation of the NOS pathway, leading to the release of NO from neurons (Pelligrino et al., 1999) and endothelial cells (Najarian et al., 2000; Peebles et al., 2008). When the NO pathway is blocked, the vasodilatory response is reduced by 36–94% depending on the inhibitor used, hypercapnia levels and species (Iadecola and Zhang, 1994). While some studies have shown that inhibiting the endothelial NO pathway or destroying endothelial cells does not abolish the CBF response to hypercapnia (Wang et al., 1994), other studies have shown that neuronal sources cannot in isolation explain the CBF response to hypercapnia (Iadecola et al., 1987, 1993; Iadecola and Zhang, 1996). This likely reflects a combined contribution of endothelial and neuronal sources or redundancy that allows one system to come online when the other fails. It is important to note, however, that there may be some important species-related differences in pathways (Najarian et al., 2000), making animal results only partly relevant to human data. Therefore, while it is currently unclear whether these two responses are truly equivalent, there are clear similarities between them, lending validity to the use of hypercapnia-based CVR as a model for the vascular component of neurovascular coupling.

## The Role of CVR in Resting-State fMRI

### What Is Resting-State fMRI?

Functional MRI in the resting state (rs-fMRI), particularly based on the BOLD signal, has been extensively used to measure functional connectivity in the brain. The use of the BOLD signal for resting-state imaging largely began with the seminal discovery of resting-state BOLD signal-based (rs-BOLD) synchronization across brain networks (namely “resting-state functional connectivity”) by Biswal et al. (Biswal et al., 1995; Fox, 2010). Despite the undefined cognitive state of the brain in the “resting-state” and the ambiguous involvement of vascular and metabolic mechanisms underlying the BOLD signal (discussed later in this section), resting-state BOLD signal-based brain networks have been consistently revealed in numerous studies. Notably, the well-documented default-mode network (DMN), key in generating cognition, has been implicated in a wide array of neurological diseases. In recent years, resting-state BOLD-fMRI has gained considerable attention in basic and clinical neuroscience (Biswal et al., 1995; Fox, 2010) and the number of publications using the resting-state BOLD contrast has seen exponential growth. This remarkable growth is attributable to the ability of rs-BOLD studies to bypass the hurdles of task performance and behavioral evaluations in assessing brain function, opening a new attractive avenue for neuroimaging research in pediatrics, aging, and a variety of neurologic and psychiatric diseases. A comprehensive description of the applications of rs-BOLD signals can be found in recent reviews (Fox, 2010).

Functional connectivity is the main reason for the popularity of resting-state fMRI. First-level resting-state functional connectivity is generally computed using seed-based correlation or data-driven approaches. In the model-based seed-correlation analysis (Biswal et al., 1995; Van Dijk et al., 2010), connectivity is defined as the correlation between the seed rs-fMRI signal time series and those of other brain voxels or regions. Data-driven methods typically use principal- or independent component analysis (PCA and ICA, respectively) to identify brain networks. While the seed-based approach is constrained by model assumptions and *a priori* hypotheses, model-free data-driven methods are more challenging to use in group-analyses due to higher variability in the network components identified. Seed- and data-driven approaches yield largely similar spatial patterns (although with differing spatial extents), and both methods can be used to determine connectivity magnitude. However, due to the linear assumptions related to the model-based connectivity methods, it is easier to intuite the influence of physiological metrics on resting-state functional connectivity. After all, there is a well-defined relationship between the correlation coefficient and the rs-fMRI signal amplitude, which is, in turn, describable by the steady-state fMRI signal model as introduced earlier. With the adoption of higher-level functional connectivity measures (those derived from the first-level metrics, such as centrality and hubness, to name a few), the effect of physiological biases may not seem obvious, but it is all the more important to understand them at more abstract levels of analysis.

### The Role of CVR in Resting-State fMRI

The rs-fMRI technique, while immensely popular, has been limited by a lack of a fundamental physiological understanding of the underlying rs-fMRI BOLD signal (Leopold and Maier, 2012). The BOLD signal is only an indirect measure of neuronal activity and is inherently modulated by both neuronal activity and vascular physiology (Biswal et al., 2007; Kannurpatti et al., 2008; Biswal and Kannurpatti, 2009; Tong and Frederick, 2010). Currently, the respective contributions of these factors to resting-state BOLD are still unknown. This knowledge gap leads to great challenges for data interpretation in clinical scenarios, whereby these contributions are often altered. The literature suggests that BOLD-based fMRI signal is fundamentally modulated by local vascular physiology (Carusone et al., 2002; Kannurpatti et al., 2010; Liu, 2013).

**FIGURE 1:**
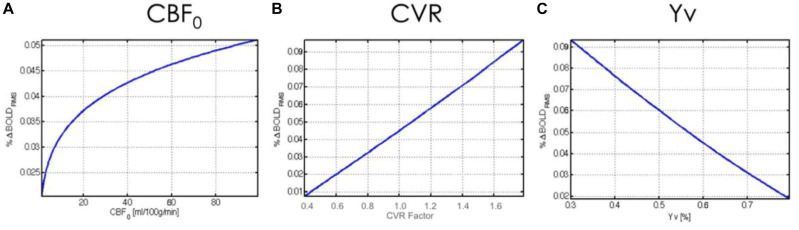
Theoretical relationship between rs-fMRI signal amplitude and physiological variables. The BOLD fMRI fluctuation amplitude (%BOLD_RMS_) is plotted against baseline **(A)** cerebral blood flow (CBF_0_), **(B)** cerebrovascular reactivity (CVR), and **(C)** venous blood oxygenation (Yv). Figure reproduced from Chu et al. (2018) with permission from Elsevier.

Previous work on the biophysical origins of the rs-fMRI signal suggests that the steady-state BOLD model (Davis et al., 1998; Hoge et al., 1999a), as outlined in the previous section, is a reasonable framework for understanding the neurovascular underpinnings of the resting BOLD effect. CVR is known to covary with the BOLD response to neuronal activation (Stefanovic et al., 2006). Specifically, reduced vascular responsiveness has been associated with reduced BOLD activation amplitude as well as a slowing down in the BOLD response dynamics (Behzadi and Liu, 2005; Rack-Gomer and Liu, 2012), setting the stage for our study of the effect of CVR on the rs-fMRI signal. Indeed, CVR is a major factor determining the hemodynamic response to neuronal activity, which in turn modulates rs-fcMRI signal amplitude [see review (Liu, 2013) and Figure 1]. As a result, CVR is expected to drive the amplitude of resting BOLD signal fluctuations (RSFA). Indeed, hypercapnia, which elevates basal CBF and oxygenation while reducing CVR (Cohen et al., 2002), has been shown to reduce the amplitude of resting-state BOLD signals (Biswal et al., 1997; Xu et al., 2011), consistent with predictions based on the BOLD signal model. Although hypercapnia has also been shown to reduce the power of the alpha-rhythm (Xu et al., 2011), CVR is likely to also play a key role in modulating the RSFA in this context. As an extension, in a study of 335 healthy older adults (Tsvetanov et al., 2015), it was found that the effects of aging on the RSFA were mediated by cardiovascular factors such as heart rate.

Interestingly, another representation of the rs-fMRI signal fluctuation, the amplitude of low-frequency fluctuations (ALFF), is typically interpreted as a neuronal measure (Zou et al., 2008; Jia et al., 2020). It is important to realize that the RSFA and ALFF are directly proportional to each other and that it is illogical to interpret the same quantity in two different ways. Furthermore, the fractional ALFF (fALFF) amounts to the RSFA normalized by the per-voxel signal variance instead of the mean (Zou et al., 2008; Jia et al., 2020). This measure would be influenced by both the neuronal signal content at the SNR at each voxel, making the physiological interpretation more tortuous.

However, the relationship between the RSFA and functional connectivity is more subtle than linear. The hemodynamic response determines the BOLD signal amplitude and subsequently the rs-fMRI BOLD SNR; different signal SNRs will in turn lead to different connectivity measurements (Liu, 2013). Such biases may obscure the meaning of rs-fMRI functional connectivity measurements (Golestani et al., 2016; Chu et al., 2018), which are modulated by the RSFA (Rack-Gomer and Liu, 2012; Tak et al., 2015).

In our previous work (Golestani et al., 2016), we demonstrated the extent of this modulation, as well as uncovered the effect of CVR modulation on rs-fMRI functional connectivity. Across the group, rs-fMRI functional connectivity of the motor network also depends significantly on the baseline capnic state, with the hypocapnic baseline associated with the highest connectivity values, and hypercapnic baseline associated with the lowest connectivity. The latter finding is in agreement with early data from Biswal et al. (1997) and from more recent data from Xu et al. (2011). This association, however, is not consistent across the brain. The recent work by Lewis et al. (2020) extends this work to dynamic functional-connectivity analyses and over 42 functional networks. It was found that network connectivity is generally weaker during vasodilation, which is supported by previous research (Biswal et al., 1997; Golestani et al., 2016).

**FIGURE 2:**
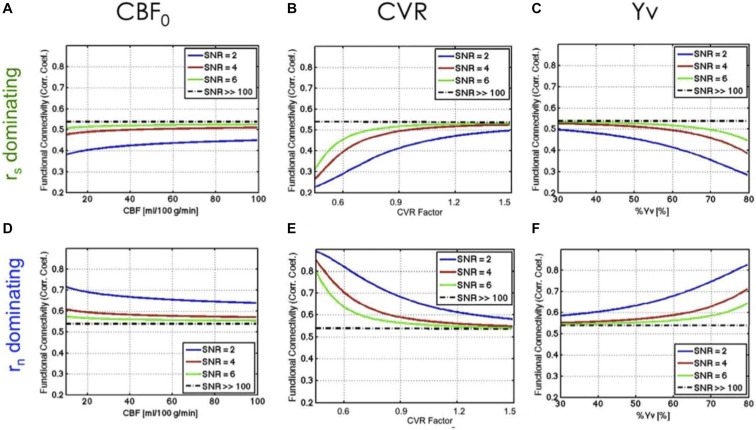
Theoretical relationship between rs-fMRI functional connectivity and physiological variables. The dependence of function connectivity (FC) on all three physiological variables (CBF_0_, CVR, and Yv) is driven by the signal-to-noise ratio (SNR) and by the balance between signal-driven (r_*s*_) **(A–C)** and noise-driven correlations (r_n_) **(D–F)**. A lower SNR leads to more emphasized dependence of FC on baseline physiology. Figure reproduced from Chu et al. (2018) with permission from Elsevier.

As follow-up work (Chu et al., 2018), instead of modulating CVR within individuals, we compared fcMRI and CVR across different individuals. There was observable inter-subject CVR variation even amongst healthy young adults, as well as a distribution of functional connectivity values. In this work, we used the steady-state BOLD model to make predictions about functional connectivity given CVR (as well as CBF and SO_2_) (Figure 2). We further used these predictions to help interpret the empirical data, with the objective of improving our understanding of the possible origins of inter-subject variations in rs-fMRI functional connectivity. However, the characterization of vascular biases on rs-fMRI metrics remains scarce in current literature.

Given the above, the open question is how to normalize or calibrate the vascular effects from rs-fMRI measures, which is especially used to study neuronal markers (Champagne et al., 2020; Coverdale et al., 2020; Tsvetanov et al., 2020). As an example of the application of a normalization approach, Xu et al. (2011) normalized the resting-state BOLD amplitudes in the default-mode network by the task-related BOLD responses in the visual cortex, as CO_2_ challenges were observed not to alter the RSFA in the visual cortex. More recently, following the findings in Chu et al. (2018) and Champagne et al. (2020) normalized functional connectivity by local CBF and observed a reduction in connectivity differences between healthy and patient groups. This is in line with observations by Garrett et al. (2017), who also observed age-related differences in RSFA to be reduced when normalized by vascular physiology. However, it is likely that the fcMRI-CVR association is mechanical (driven by local CVR and the local vessel composition) and coincidental (driven by the relationship of both to neuronal health). Hence, adequate correction of vascular bias remains an open challenge.

### Quantifying CVR Using Resting-State fMRI

Resting-state fMRI offers a unique opportunity to glean CVR information without the need for respiratory challenges. This type of “unconstrained” or “task-free” CVR protocol does not require cooperation from participants, and is thus a promising direction of research that will likely broaden the accessibility of CVR mapping to clinical researchers (Chen, 2018).

The RSFA was initially introduced as a vascular scaling factor for task-based BOLD responses by Kannurpatti et al. (2011), as it was used to scale fMRI task responses in the study of aging. Since then, resting-state methods that do not require CO_2_ perturbation have flourished (Golestani et al., 2016; Jahanian et al., 2017; Liu et al., 2017). Notably, Kannurpatti et al. (2014) reported a comparison of the resting-state fMRI fluctuation amplitude (voxel-wise temporal standard deviation) as a CVR surrogate. Liu et al. (2017) introduced a method that uses the low-frequency range of the rs-fMRI signal, regressed against the global-signal, to generate a qualitative CVR estimate. In the same year, Jahanian et al. (2017) introduced the concept of using either the voxel-wise regression coefficients with cerebrospinal fluid signal (measured using rs-fMRI) or using the voxel-wise coefficient of variation to approximate CVR. The former is more akin to the approach of Liu et al. (2017) while the latter follows the logic of the method by Kannurpatti et al. (2014) In the meantime, the Liu method has also been demonstrated in delineating global and local CVR deficits (in Moyamoya disease and acute stroke, respectively) (Taneja et al., 2019).

While all of these methods have demonstrated correlations with CO_2_-based CVR measures, they provide qualitative measures of CVR, which complicates inter-subject comparisons, quantitative CVR mapping techniques using rs-fMRI remain scarce. The only such technique takes advantage of spontaneous fluctuations in end-tidal CO_2_ while eliminating the effects of heart-rate and respiratory-volume variability on the fMRI signal (Golestani et al., 2016). Once the CO_2_-related BOLD signal is isolated, a deconvolution is performed between the resting-state fMRI and CO_2_ time courses, and the area under the response function is by definition the quantitative CVR. This approach echoes the derivation of the CO_2_ response function using a series of breathing maneuvers, including deep breathing, rapid breathing and free breathing (Vogt et al., 2011). The derivation of the whole response function instead of a simple CVR amplitude provides the opportunity to extract temporal CVR features.

As in the case of calibrated fMRI, one should be aware of spatial heterogeneities and non-linearities in the flow and BOLD response to CO_2_. For instance, the use of CVR to normalize rs-fMRI metrics is only a valid approach if one can assume that the CVR response obtained in different individuals and brain regions are taken from the same linear regime.

### Other Vascular Sources of Resting-State fMRI

rBeyond the magnitude of CVR, the dynamic features of the fMRI response function that are available through this method can also provide useful information. A slowing of the CVR response has been shown to characterize vascular lesions (Poublanc et al., 2015), adding a dimension to the utility of CVR mapping. Indeed, differences between young and older adults have been demonstrated using simply the temporal features of the resting CO_2_ response function (Esmaelbeigi and Chen, 2019). It is further shown that by accounting for the temporal shifts in the BOLD CO2 response, improved CVR estimates can be obtained (Yao et al., 2021). Furthermore, this CO_2_-related shift structure can be associated with the lag structure that naturally occurs in the rs-fMRI signal (Tong and Frederick, 2014; Aso et al., 2019), and can be capitalized to extract additional vascular information without CO_2_ challenges.

Respiratory-volume variability (RVT) also modulates the rs-fMRI signal (Birn et al., 2006), and a potential relationship between CO_2_ and RVT has previously been identified (Vogt et al., 2011). This was extended to the convolution of the RVT with its fMRI response function (Chang and Glover, 2009). Thus, RVT can also be considered as a potential physiological marker to yield a CVR measure, a possible future research direction.

The CO_2_ response has also been reported to exhibit network structure that coincide with that of conventional rs-fMRI functional networks (Bright et al., 2020). Cardiac pulsatility, which entrains the autonomic nervous system, is also known to contribute to the rs-fMRI signal in a spatially specific manner (Shmueli et al., 2007; Chang et al., 2009; Shokri-Kojori et al., 2018; Attarpour et al., 2021). In fact, a substantial portion of the rs-fMRI signal may stem from cardiogenic vascular oscillations, and such signals can interact with respiration and CO2 in a biofeedback loop (Attarpour et al., 2021). Respiratory and cardiac-related physiological networks have recently been documented in detail (Chen et al., 2020).

## Current Challenges and Future Directions

Cerebrovascular reactivity has been found to associate with cerebral autoregulation, which is assessed as the phase shift between arterial blood pressure and venous blood flow changes (Carrera et al., 2009). This association is observed in vascular pathologies (Chen et al., 2014), and can influence the degree to which the vasculature responds to CO_2_. Thus, the use of CVR in calibrated BOLD and resting BOLD can lead to biased results in certain patient populations.

Novel accelerated imaging acquisitions can enhance the power of calibrated and resting-state fMRI. Specifically, the use of multi-echo BOLD in conjunction with simultaneous multi-slice acceleration has led to improved isolation of BOLD from noise contributions in the task and resting fMRI alike (Olafsson et al., 2015) as well as to more robust CVR estimates (Cohen and Wang, 2019). The ability to accelerate acquisitions using slice acceleration has also led to improved efficiency in terms of sensitivity per unit time for capturing the BOLD response (Todd et al., 2017). Moreover, a slice acceleration factor of up to four can be achieved without sacrificing rs-fMRI data quality (Preibisch et al., 2015), with the time savings opening up possibilities for CVR scans to be included in a typical scan session.

Despite the wide use of calibrated fMRI, the fundamental accuracy of the CO_2_-calibrated BOLD model remains to be validated under different conditions (Hoge, 2012; Blockley et al., 2015; Chen, 2019). While the basic principle of CVR involvement in the BOLD response remains valid, the extent to which different vascular compartments are involved, and the resultant BOLD signal evolution, continue to be refined using electrophysiological recordings (Shmuel et al., 2006; Liu et al., 2010; Sanganahalli et al., 2016), near-infrared spectroscopy (Boas et al., 2003; Gagnon et al., 2012), and two-photon microscopy (Gagnon et al., 2016). In the latter work, the interpretation of the Davis model and the calibration process is revised using microscopy-informed Monte Carlo simulations of the BOLD signal. In rs-fMRI, the desire to isolate the neuronal from the vascular fluctuations has led to the involvement of electroencephalography (EEG) (Chang et al., 2013; Liu et al., 2014) and glucose positron emission tomography (PET) (Thompson et al., 2016). While the approach of normalizing rs-fMRI metrics by CVR may be an oversimplification, it is a viable first step in extracting the neuronal information from fMRI alone.

## Conclusion

Cerebrovascular reactivity is important for the interpretation of both task-based and resting-state fMRI results. The need to incorporate CVR into fMRI data interpretation is increasingly recognized, but more accessible ways of mapping CVR are necessary for wide adoption.

## Author Contributions

All authors listed have made a substantial, equal, direct and intellectual contribution to the work, and approved it for publication.

## Conflict of Interest

The authors declare that the research was conducted in the absence of any commercial or financial relationships that could be construed as a potential conflict of interest.

## References

[B1] Ances B. Vaida F. Ellis R. Buxton R. (2011). Test-retest stability of calibrated BOLD-fMRI in HIV- and HIV+ subjects. *Neuroimage* 54 2156–2162. 10.1016/j.neuroimage.2010.09.081 20932922 PMC3229916

[B2] Aso T. Urayama S. Fukuyama H. Murai T. (2019). Axial variation of deoxyhemoglobin density as a source of the low-frequency time lag structure in blood oxygenation level-dependent signals. *PLoS One* 14:e0222787. 10.1371/journal.pone.0222787 31545839 PMC6756514

[B3] Attarpour A. Ward J. Chen J. J. (2021). Vascular origins of low-frequency oscillations in the cerebrospinal fluid signal in resting-state fMRI: validation using photoplethysmography. *Hum. Brain Mapp.* 1 1–2. in press, 10.1002/hbm.25392 PMC8090775 33638224

[B4] Attwell D. Buchan A. M. Charpak S. Lauritzen M. Macvicar B. A. Newman E. A. (2010). Glial and neuronal control of brain blood flow. *Nature* 468 232–243. 10.1038/nature09613 21068832 PMC3206737

[B5] Bandettini P. A. Wong E. C. (1997). A hypercapnia-based normalization method for improved spatial localization of human brain activation with fMRI. *NMR Biomed.* 10 197–203. 10.1002/(sici)1099-1492(199706/08)10:4/5<197::aid-nbm466>3.0.co;2-s 9430348

[B6] Barratt-Boyes B. G. Wood E. H. (1957). The oxygen saturation of blood in the venae cavae, right-heart chambers, and pulmonary vessels of healthy subjects. *J. Lab. Clin. Med.* 50 93–106. 13439270

[B7] Behzadi Y. Liu T. T. (2005). An arteriolar compliance model of the cerebral blood flow response to neural stimulus. *Neuroimage* 25 1100–1111. 10.1016/j.neuroimage.2004.12.057 15850728

[B8] Birn R. M. Diamond J. B. Smith M. A. Bandettini P. A. (2006). Separating respiratory-variation-related fluctuations from neuronal-activity-related fluctuations in fMRI. *Neuroimage* 31 1536–1548. 10.1016/j.neuroimage.2006.02.048 16632379

[B9] Biswal B. Hudetz A. G. Yetkin F. Z. Haughton V. M. Hyde J. S. (1997). Hypercapnia reversibly suppresses low-frequency fluctuations in the human motor cortex during rest using echo-planar MRI. *J. Cereb. Blood Flow Metab.* 17 301–308. 10.1097/00004647-199703000-00007 9119903

[B10] Biswal B. Zerrin Yetkin F. Haughton V. M. Hyde J. S. (1995). Functional connectivity in the motor cortex of resting human brain using echo-planar mri. *Magn. Reson. Med.* 34 537–541. 10.1002/mrm.1910340409 8524021

[B11] Biswal B. B. Kannurpatti S. S. (2009). Resting-state functional connectivity in animal models: modulations by exsanguination. *Methods Mol. Biol.* 489 255–274. 10.1007/978-1-59745-543-5_12 18839096 PMC10671844

[B12] Biswal B. B. Kannurpatti S. S. Rypma B. (2007). Hemodynamic scaling of fMRI-BOLD signal: validation of low-frequency spectral amplitude as a scalability factor. *Magn. Reson. Imaging* 25 1358–1369. 10.1016/j.mri.2007.03.022 17482411 PMC2701471

[B13] Blockley N. P. Griffeth V. E. M. Stone A. J. Hare H. V. Bulte D. P. (2015). Sources of systematic error in calibrated BOLD based mapping of baseline oxygen extraction fraction. *Neuroimage* 122 105–113. 10.1016/j.neuroimage.2015.07.059 26254114

[B14] Boas D. A. Strangman G. Culver J. P. Hoge R. D. Jasdzewski G. Poldrack R. A. (2003). Can the cerebral metabolic rate of oxygen be estimated with near-infrared spectroscopy? *Phys. Med. Biol.* 48 2405–2418. 10.1088/0031-9155/48/15/311 12953906

[B15] Bright M. G. Bulte D. P. Jezzard P. Duyn J. H. (2009). An alternative technique for measuring cerebrovascular reactivity; comparing cued deep breathing hypocapnia with inspiration of carbon dioxide. *Proc. Intl. Soc. Mag. Reson. Med.* 17 1634.

[B16] Bright M. G. Whittaker J. R. Driver I. D. Murphy K. (2020). Vascular physiology drives functional brain networks. *Neuroimage* 217:116907. 10.1016/j.neuroimage.2020.116907 32387624 PMC7339138

[B17] Bulte D. P. Kelly M. Germuska M. Xie J. Chappell M. A. Okell T. W. (2012). Quantitative measurement of cerebral physiology using respiratory-calibrated MRI. *Neuroimage* 60 582–591. 10.1016/j.neuroimage.2011.12.017 22209811 PMC7100043

[B18] Carrera E. Lee L. K. Giannopoulos S. Marshall R. S. (2009). Cerebrovascular reactivity and cerebral autoregulation in normal subjects. *J. Neurol. Sci.* 285 191–194. 10.1016/j.jns.2009.06.041 19608202

[B19] Carusone L. M. Srinivasan J. Gitelman D. R. Mesulam M.-M. Parrish T. B. (2002). Hemodynamic response changes in cerebrovascular disease: implications for functional MR imaging. *AJNR Am. J. Neuroradiol.* 23 1222–1228. 12169483 PMC8185726

[B20] Champagne A. A. Coverdale N. S. Ross A. Chen Y. Murray C. I. Dubowitz D. (2020). Multi-modal normalization of resting-state using local physiology reduces changes in functional connectivity patterns observed in mTBI patients. *Neuroimage Clin.* 26:102204. 10.1016/j.nicl.2020.102204 32058317 PMC7013121

[B21] Chang C. Cunningham J. P. Glover G. H. (2009). Influence of heart rate on the BOLD signal: the cardiac response function. *Neuroimage* 44 857–869. 10.1016/j.neuroimage.2008.09.029 18951982 PMC2677820

[B22] Chang C. Glover G. H. (2009). Relationship between respiration, end-tidal CO2, and BOLD signals in resting-state fMRI. *Neuroimage* 47 1381–1393. 10.1016/j.neuroimage.2009.04.048 19393322 PMC2721281

[B23] Chang C. Liu Z. Chen M. C. Liu X. Duyn J. H. (2013). EEG correlates of time-varying BOLD functional connectivity. *Neuroimage* 72 227–236. 10.1016/j.neuroimage.2013.01.049 23376790 PMC3602157

[B24] Chen J. Liu J. Xu W.-H. Xu R. Hou B. Cui L.-Y. (2014). Impaired dynamic cerebral autoregulation and cerebrovascular reactivity in middle cerebral artery stenosis. *PLoS One* 9:e88232. 10.1371/journal.pone.0088232 24505442 PMC3913771

[B25] Chen J. E. Lewis L. D. Chang C. Tian Q. Fultz N. E. Ohringer N. A. (2020). Resting-state “physiological networks.”. *Neuroimage* 213:116707. 10.1016/j.neuroimage.2020.116707 32145437 PMC7165049

[B26] Chen J. J. (2018). Cerebrovascular-reactivity mapping using MRI: considerations for Alzheimer’s disease. *Front. Aging. Neurosci.* 10:170. 10.3389/fnagi.2018.00170 29922153 PMC5996106

[B27] Chen J. J. (2019). Functional MRI of brain physiology in aging and neurodegenerative diseases. *Neuroimage* 187 209–225. 10.1016/j.neuroimage.2018.05.050 29793062

[B28] Chen J. J. Pike G. B. (2009). BOLD-specific cerebral blood volume and blood flow changes during neuronal activation in humans. *NMR Biomed.* 22 1054–1062. 10.1002/nbm.1411 19598180

[B29] Chen J. J. Pike G. B. (2010a). Global cerebral oxidative metabolism during hypercapnia and hypocapnia in humans: implications for BOLD fMRI. *J. Cereb. Blood Flow Metab.* 30 1094–1099. 10.1038/jcbfm.2010.42 20372169 PMC2949195

[B30] Chen J. J. Pike G. B. (2010b). MRI measurement of the BOLD-specific flow–volume relationship during hypercapnia and hypocapnia in humans. *Neuroimage*. 53 383–391. 10.1016/j.neuroimage.2010.07.003 20624474

[B31] Chiarelli P. A. Bulte D. P. Wise R. Gallichan D. Jezzard P. (2007). A calibration method for quantitative BOLD fMRI based on hyperoxia. *Neuroimage* 37 808–820. 10.1016/j.neuroimage.2007.05.033 17632016

[B32] Chu P. P. W. Golestani A. M. Kwinta J. B. Khatamian Y. B. Chen J. J. (2018). Characterizing the modulation of resting-state fMRI metrics by baseline physiology. *Neuroimage*. 173 72–87. 10.1016/j.neuroimage.2018.02.004 29452265

[B33] Cohen A. D. Wang Y. (2019). Improving the assessment of breath-holding induced cerebral vascular reactivity using a multiband multi-echo ASL/BOLD sequence. *Sci. Rep.* 9:5079. 30911056 10.1038/s41598-019-41199-w PMC6434035

[B34] Cohen E. R. Ugurbil K. Kim S. G. (2002). Effect of basal conditions on the magnitude and dynamics of the blood oxygenation level-dependent fMRI response. *J. Cereb. Blood Flow Metab.* 22 1042–1053. 10.1097/00004647-200209000-00002 12218410

[B35] Coverdale N. S. Fernandez-Ruiz J. Champagne A. A. Mark C. I. Cook D. J. (2020). Co-localized impaired regional cerebrovascular reactivity in chronic concussion is associated with BOLD activation differences during a working memory task. *Brain Imaging Behav.* 14 2438–2449. 10.1007/s11682-019-00194-5 31903527

[B36] Cukic V. (2014). The changes of arterial blood gases in COPD during four-year period. *Mediev. Archaeol.* 68 14–18. 10.5455/medarh.2014.68.14-18 24783904 PMC4272457

[B37] Davis T. L. Kwong K. K. Weisskoff R. M. Rosen B. R. (1998). Calibrated functional MRI: mapping the dynamics of oxidative metabolism. *Proc. Natl. Acad. Sci. U. S. A.* 95 1834–1839. 10.1073/pnas.95.4.1834 9465103 PMC19199

[B38] De Vis J. B. Hendrikse J. Bhogal A. Adams A. Kappelle L. J. Petersen E. T. (2015). Age-related changes in brain hemodynamics; a calibrated MRI study. *Hum. Brain Mapp.* 36 3973–3987. 10.1002/hbm.22891 26177724 PMC6869092

[B39] Driver I. D. Wise R. G. Murphy K. (2017). Graded hypercapnia-calibrated BOLD: beyond the Iso-metabolic hypercapnic assumption. *Front. Neurosci.* 11:276. 10.3389/fnins.2017.00276 28572755 PMC5435758

[B40] Duffin J. Sobczyk O. Crawley A. Poublanc J. Venkatraghavan L. Sam K. (2017). The role of vascular resistance in BOLD responses to progressive hypercapnia. *Hum. Brain Mapp.* 38 5590–5602. 10.1002/hbm.23751 28782872 PMC6866756

[B41] Esmaelbeigi A. Chen J. J. (2019). The effect of age on resting state fMRI carbon dioxide response function. *Proc. Organ. Hum. Brain Mapp.* 2019:1403.

[B42] Faraci F. M. Brian J. E. Jr. (1994). Nitric oxide and the cerebral circulation. *Stroke* 25 692–703. 10.1161/01.str.25.3.692 7510430

[B43] Fathi A. R. Yang C. Bakhtian K. D. Qi M. Lonser R. R. Pluta R. M. (2011). Carbon dioxide influence on nitric oxide production in endothelial cells and astrocytes: cellular mechanisms. *Brain Res.* 1386 50–57. 10.1016/j.brainres.2011.02.066 21362408 PMC3073030

[B44] Fierstra J. Sobczyk O. Battisti-Charbonney A. Mandell D. M. Poublanc J. Crawley A. P. (2013). Measuring cerebrovascular reactivity: what stimulus to use? *J. Physiol.* 591 5809–5821. 10.1113/jphysiol.2013.259150 24081155 PMC3872753

[B45] Fox M. D. (2010). Clinical applications of resting state functional connectivity. *Front. Syst. Neurosci.* 4:19. 10.3389/fnsys.2010.00019 20592951 PMC2893721

[B46] Gagnon L. Sakadžiæ S. Lesage F. Pouliot P. Dale A. M. Devor A. (2016). Validation and optimization of hypercapnic-calibrated fMRI from oxygen-sensitive two-photon microscopy. *Philos. Trans. R. Soc. Lond. B Biol. Sci.* 371:20150359. 10.1098/rstb.2015.0359 27574311 PMC5003859

[B47] Gagnon L. Yücel M. A. Dehaes M. Cooper R. J. Perdue K. L. Selb J. (2012). Quantification of the cortical contribution to the NIRS signal over the motor cortex using concurrent NIRS-fMRI measurements. *Neuroimage* 59 3933–3940. 10.1016/j.neuroimage.2011.10.054 22036999 PMC3279595

[B48] Garrett D. D. Lindenberger U. Hoge R. D. Gauthier C. J. (2017). Age differences in brain signal variability are robust to multiple vascular controls. *Sci. Rep.* 7:10149. 28860455 10.1038/s41598-017-09752-7 PMC5579254

[B49] Gauthier C. J. Fan A. P. (2019). BOLD signal physiology: models and applications. *Neuroimage* 187 116–127. 10.1016/j.neuroimage.2018.03.018 29544818

[B50] Gauthier C. J. Hoge R. D. (2012). Magnetic resonance imaging of resting OEF and CMRO2 using a generalized calibration model for hypercapnia and hyperoxia. *Neuroimage* 60 1212–1225. 10.1016/j.neuroimage.2011.12.056 22227047

[B51] Gauthier C. J. Hoge R. D. (2013). A generalized procedure for calibrated MRI incorporating hyperoxia and hypercapnia. *Hum. Brain Mapp.* 34 1053–1069. 10.1002/hbm.21495 23015481 PMC6870118

[B52] Gauthier C. J. Madjar C. Desjardins-Crépeau L. Bellec P. Bherer L. Hoge R. D. (2012). Age dependence of hemodynamic response characteristics in human functional magnetic resonance imaging. *Neurobiol. Aging.* 34 1469–1485. 10.1016/j.neurobiolaging.2012.11.002 23218565

[B53] Girouard H. Iadecola C. (2006). Neurovascular coupling in the normal brain and in hypertension, stroke, and Alzheimer disease. *J. Appl. Physiol.* 100 328–335. 10.1152/japplphysiol.00966.2005 16357086

[B54] Golestani A. M. Kwinta J. B. Strother S. C. Khatamian Y. B. Chen J. J. (2016). The association between cerebrovascular reactivity and resting-state fMRI functional connectivity in healthy adults: the influence of basal carbon dioxide. *Neuroimage* 132 301–313. 10.1016/j.neuroimage.2016.02.051 26908321 PMC5148617

[B55] Griffeth V. E. M. Buxton R. B. (2011). A theoretical framework for estimating cerebral oxygen metabolism changes using the calibrated-BOLD method: modeling the effects of blood volume distribution, hematocrit, oxygen extraction fraction, and tissue signal properties on the BOLD signal. *Neuroimage* 58 198–212. 10.1016/j.neuroimage.2011.05.077 21669292 PMC3187858

[B56] Halani S. Kwinta J. B. Golestani A. M. Khatamian Y. B. Chen J. J. (2015). Comparing cerebrovascular reactivity measured using BOLD and cerebral blood flow MRI: the effect of basal vascular tension on vasodilatory and vasoconstrictive reactivity. *Neuroimage* 110 110–123. 10.1016/j.neuroimage.2015.01.050 25655446 PMC5167565

[B57] Hardie J. A. Vollmer W. M. Buist A. S. Ellingsen I. Mørkve O. (2004). Reference values for arterial blood gases in the elderly. *Chest* 125 2053–2060. 10.1378/chest.125.6.2053 15189921

[B58] Hoge R. D. (2012). Calibrated fMRI. *Neuroimage* 62 930–937. 10.1016/j.neuroimage.2012.02.022 22369993

[B59] Hoge R. D. Atkinson J. Gill B. Crelier G. R. Marrett S. Pike G. B. (1999a). Investigation of BOLD signal dependence on cerebral blood flow and oxygen consumption: the deoxyhemoglobin dilution model. *Magn. Reson. Med.* 42 849–863. 10.1002/(sici)1522-2594(199911)42:5<849::aid-mrm4>3.0.co;2-z 10542343

[B60] Hoge R. D. Atkinson J. Gill B. Crelier G. R. Marrett S. Pike G. B. (1999b). Linear coupling between cerebral blood flow and oxygen consumption in activated human cortex. *Proc. Natl. Acad. Sci. U. S. A.* 96 9403–9408. 10.1073/pnas.96.16.9403 10430955 PMC17795

[B61] Hülsmann W. C. Dubelaar M. L. (1988). Aspects of fatty acid metabolism in vascular endothelial cells. *Biochimie* 70 681–686. 10.1016/0300-9084(88)90253-2 3139085

[B62] Iadecola C. (2017). The neurovascular unit coming of age: a journey through neurovascular coupling in health and disease. *Neuron* 96 17–42. 10.1016/j.neuron.2017.07.030 28957666 PMC5657612

[B63] Iadecola C. Arneric S. P. Baker H. D. Tucker L. W. Reis D. J. (1987). Role of local neurons in cerebrocortical vasodilation elicited from cerebellum. *Am. J. Physiol.* 252 R1082–R1091. 3591980 10.1152/ajpregu.1987.252.6.R1082

[B64] Iadecola C. Zhang F. (1994). Nitric oxide-dependent and -independent components of cerebrovasodilation elicited by hypercapnia. *Am. J. Physiol.* 266 R546–R552. 7511352 10.1152/ajpregu.1994.266.2.R546

[B65] Iadecola C. Zhang F. (1996). Permissive and obligatory roles of NO in cerebrovascular responses to hypercapnia and acetylcholine. *Am. J. Physiol.* 271 R990–R1001. 8897992 10.1152/ajpregu.1996.271.4.R990

[B66] Iadecola C. Zhang F. Xu X. (1993). Role of nitric oxide synthase-containing vascular nerves in cerebrovasodilation elicited from cerebellum. *Am. J. Physiol.* 264 R738–R746. 7682793 10.1152/ajpregu.1993.264.4.R738

[B67] Jahanian H. Christen T. Moseley M. E. Pajewski N. M. Wright C. B. Tamura M. K. (2017). Measuring vascular reactivity with resting-state blood oxygenation level-dependent (BOLD) signal fluctuations: a potential alternative to the breath-holding challenge? *J. Cerebral Blood Flow Metab.* 37 2526–2538. 10.1177/0271678x16670921 27683452 PMC5531349

[B68] Jain V. Langham M. C. Floyd T. F. Jain G. Magland J. F. Wehrli F. W. (2011). Rapid magnetic resonance measurement of global cerebral metabolic rate of oxygen consumption in humans during rest and hypercapnia. *J. Cereb. Blood Flow Metab.* 31 1504–1512. 10.1038/jcbfm.2011.34 21505481 PMC3137470

[B69] Jia X.-Z. Sun J.-W. Ji G.-J. Liao W. Lv Y.-T. Wang J. (2020). Percent amplitude of fluctuation: a simple measure for resting-state fMRI signal at single voxel level. *PLoS One* 15:e0227021. 10.1371/journal.pone.0227021 31914167 PMC6948733

[B70] Kannurpatti S. S. Biswal B. B. Kim Y. R. Rosen B. R. (2008). Spatio-temporal characteristics of low-frequency BOLD signal fluctuations in isoflurane-anesthetized rat brain. *Neuroimage* 40 1738–1747. 10.1016/j.neuroimage.2007.05.061 18339559 PMC10671857

[B71] Kannurpatti S. S. Motes M. A. Biswal B. B. Rypma B. (2014). Assessment of unconstrained cerebrovascular reactivity marker for large age-range FMRI studies. *PLoS One* 9:e88751. 10.1371/journal.pone.0088751 24551151 PMC3923811

[B72] Kannurpatti S. S. Motes M. A. Rypma B. Biswal B. B. (2010). Neural and vascular variability and the fMRI-BOLD response in normal aging. *Magn. Reson. Imaging* 28 466–476. 10.1016/j.mri.2009.12.007 20117893 PMC2860003

[B73] Kannurpatti S. S. Motes M. A. Rypma B. Biswal B. B. (2011). BOLD signal change: minimizing vascular contri- butions by resting-state-fluctuation-of-amplitude scaling. *Hum. Brain Mapp.* 32 1125–1140. 10.1002/hbm.21097 20665721 PMC3310892

[B74] Komori M. Takada K. Tomizawa Y. Nishiyama K. Kawamata M. Ozaki M. (2007). Permissive range of hypercapnia for improved peripheral microcirculation and cardiac output in rabbits. *Crit. Care Med.* 35 2171–2175. 10.1097/01.ccm.0000281445.77223.31 17855833

[B75] Lajoie I. Nugent S. Debacker C. Dyson K. Tancredi F. B. Badhwar A. (2017). Application of calibrated fMRI in Alzheimer’s disease. *Neuroimage Clin.* 15 348–358. 28560160 10.1016/j.nicl.2017.05.009 PMC5443910

[B76] Leopold D. A. Maier A. (2012). Ongoing physiological processes in the cerebral cortex. *Neuroimage* 62 2190–2200. 10.1016/j.neuroimage.2011.10.059 22040739 PMC3288739

[B77] Lewis N. Lu H. Liu P. Hou X. Damaraju E. Iraji A. (2020). Static and dynamic functional connectivity analysis of cerebrovascular reactivity: An fMRI study. *Brain Behav.* 10:e01516. 32342644 10.1002/brb3.1516 PMC7303385

[B78] Liu P. Hebrank A. C. Rodrigue K. M. Kennedy K. M. Section J. Park D. C. (2013). Age-related differences in memory-encoding fMRI responses after accounting for decline in vascular reactivity. *Neuroimage* 78 415–425. 10.1016/j.neuroimage.2013.04.053 23624491 PMC3694392

[B79] Liu P. Li Y. Pinho M. Park D. C. Welch B. G. Lu H. (2017). Cerebrovascular reactivity mapping without gas challenges. *Neuroimage* 146 320–326. 10.1016/j.neuroimage.2016.11.054 27888058 PMC5321860

[B80] Liu T. T. (2013). Neurovascular factors in resting-state functional MRI. *NeuroImage* 80 339–348. 10.1016/j.neuroimage.2013.04.071 23644003 PMC3746765

[B81] Liu Z. de Zwart J. A. Chang C. Duan Q. van Gelderen P. Duyn J. H. (2014). Neuroelectrical decomposition of spontaneous brain activity measured with functional magnetic resonance imaging. *Cereb. Cortex* 24 3080–3089. 10.1093/cercor/bht164 23796947 PMC4200040

[B82] Liu Z. Rios C. Zhang N. Yang L. Chen W. He B. (2010). Linear and nonlinear relationships between visual stimuli, EEG and BOLD fMRI signals. *Neuroimage* 50 1054–1066. 10.1016/j.neuroimage.2010.01.017 20079854 PMC2841568

[B83] Mark C. I. Mazerolle E. L. Chen J. J. (2015). Metabolic and vascular origins of the BOLD effect: Implications for imaging pathology and resting-state brain function. *J. Magn. Reson. Imaging* 42 231–246. 10.1002/jmri.24786 25727523

[B84] Mazerolle P. Philouze P. Garrel R. Aubry K. Morinière S. El Bedoui S. (2018). Oncological and functional outcomes of trans-oral robotic surgery for pyriform sinus carcinoma: a french GETTEC group study. *Oral Oncol.* 86 165–170. 10.1016/j.oraloncology.2018.09.014 30409296

[B85] Mishra A. Reynolds J. P. Chen Y. Gourine A. V. Rusakov D. A. Attwell D. (2016). Astrocytes mediate neurovascular signaling to capillary pericytes but not to arterioles. *Nat. Neurosci.* 19 1619–1627. 10.1038/nn.4428 27775719 PMC5131849

[B86] Najarian T. Marrache A. M. Dumont I. Hardy P. Beauchamp M. H. Hou X. (2000). Prolonged hypercapnia-evoked cerebral hyperemia via K(+) channel- and prostaglandin E(2)-dependent endothelial nitric oxide synthase induction. *Circ. Res.* 87 1149–1156. 10.1161/01.res.87.12.1149 11110772

[B87] Olafsson V. Kundu P. Wong E. C. Bandettini P. A. Liu T. T. (2015). Enhanced identification of BOLD-like components with multi-echo simultaneous multi-slice (MESMS) fMRI and multi-echo ICA. *Neuroimage* 112 43–51. 10.1016/j.neuroimage.2015.02.052 25743045 PMC4408238

[B88] Peebles K. C. Richards A. M. Celi L. McGrattan K. Murrell C. J. Ainslie P. N. (2008). Human cerebral arteriovenous vasoactive exchange during alterations in arterial blood gases. *J. Appl. Physiol.* 105 1060–1068. 10.1152/japplphysiol.90613.2008 18617625

[B89] Pelligrino D. A. Santizo R. A. Wang Q. (1999). Miconazole represses CO(2)-induced pial arteriolar dilation only under selected circumstances. *Am. J. Physiol.* 277 H1484–H1490. 10516186 10.1152/ajpheart.1999.277.4.H1484

[B90] Pinto J. Bright M. G. Bulte D. P. Figueiredo P. (2020). Cerebrovascular reactivity mapping without gas challenges: a methodological guide. *Front. Physiol.* 11:608475. 10.3389/fphys.2020.608475 33536935 PMC7848198

[B91] Poublanc J. Crawley A. P. Sobczyk O. Montandon G. Sam K. Mandell D. M. (2015). Measuring cerebrovascular reactivity: the dynamic response to a step hypercapnic stimulus. *J. Cereb. Blood Flow Metab.* 35 1746–1756. 10.1038/jcbfm.2015.114 26126862 PMC4635229

[B92] Preibisch C. Castrillón G. J. G. Bührer M. Riedl V. (2015). Evaluation of multiband EPI acquisitions for resting state fMRI. *PLoS One* 10:e0136961. 10.1371/journal.pone.0136961 26375666 PMC4574400

[B93] Rack-Gomer A. L. Liu T. T. (2012). Caffeine increases the temporal variability of resting-state BOLD connectivity in the motor cortex. *Neuroimage* 59 2994–3002. 10.1016/j.neuroimage.2011.10.001 22032947 PMC3350816

[B94] Rancillac A. Rossier J. Guille M. Tong X.-K. Geoffroy H. Amatore C. (2006). Glutamatergic control of microvascular tone by distinct GABA neurons in the cerebellum. *J. Neurosci.* 26 6997–7006. 10.1523/jneurosci.5515-05.2006 16807329 PMC6673912

[B95] Sanganahalli B. G. Herman P. Rothman D. L. Blumenfeld H. Hyder F. (2016). Metabolic demands of neural-hemodynamic associated and disassociated areas in brain. *J. Cereb. Blood Flow Metab.* 36 1695–1707. 10.1177/0271678x16664531 27562867 PMC5076793

[B96] Shmuel A. Augath M. Oeltermann A. Logothetis N. K. (2006). Negative functional MRI response correlates with decreases in neuronal activity in monkey visual area V1. *Nat. Neurosci.* 9 569–577. 10.1038/nn1675 16547508

[B97] Shmueli K. van Gelderen P. de Zwart J. A. Horovitz S. G. Fukunaga M. Jansma J. M. (2007). Low-frequency fluctuations in the cardiac rate as a source of variance in the resting-state fMRI BOLD signal. *Neuroimage* 38 306–320. 10.1016/j.neuroimage.2007.07.037 17869543 PMC2128785

[B98] Shokri-Kojori E. Tomasi D. Volkow N. D. (2018). An autonomic network: synchrony between slow rhythms of pulse and brain resting state is associated with personality and emotions. *Cerebral Cortex* 28 3356–3371. 10.1093/cercor/bhy144 29955858 PMC6095212

[B99] Slowik J. M. Collen J. F. (2020). *Obstructive Sleep Apnea.* Treasure Island, FL: StatPearls Publishing. 29083619

[B100] Specht K. (2019). Current challenges in translational and clinical fMRI and future directions. *Front. Psychiatry* 10:924. 10.3389/fpsyt.2019.00924 31969840 PMC6960120

[B101] Stefanovic B. Schwindt W. Hoehn M. Silva A. C. (2007). Functional uncoupling of hemodynamic from neuronal response by inhibition of neuronal nitric oxide synthase. *J. Cereb. Blood Flow Metab.* 27 741–754. 10.1038/sj.jcbfm.9600377 16883353

[B102] Stefanovic B. Warnking J. M. Rylander K. M. Pike G. B. (2006). The effect of global cerebral vasodilation on focal activation hemodynamics. *Neuroimage* 30 726–734. 10.1016/j.neuroimage.2005.10.038 16337135

[B103] Tak S. Polimeni J. R. Wang D. J. J. Yan L. Chen J. J. (2015). Associations of resting-state fMRI functional connectivity with flow-BOLD coupling and regional vasculature. *Brain Connect.* 5 137–146. 10.1089/brain.2014.0299 25384681 PMC4394176

[B104] Tancredi F. B. Gauthier C. J. Madjar C. Bolar D. S. Fisher J. A. Wang D. J. J. (2012). Comparison of pulsed and pseudocontinuous arterial spin-labeling for measuring CO2-induced cerebrovascular reactivity. *J. Magn. Reson. Imaging* 36 312–321. 10.1002/jmri.23658 22544711

[B105] Taneja K. Lu H. Welch B. G. Thomas B. P. Pinho M. Lin D. (2019). Evaluation of cerebrovascular reserve in patients with cerebrovascular diseases using resting-state MRI: a feasibility study. *Magn. Reson. Imaging* 59 46–52. 10.1016/j.mri.2019.03.003 30849484 PMC6494444

[B106] Thompson G. J. Riedl V. Grimmer T. Drzezga A. Herman P. Hyder F. (2016). The whole-brain “Global” signal from resting state fMRI as a potential biomarker of quantitative state changes in glucose metabolism. *Brain Connect.* 6 435–447. 10.1089/brain.2015.0394 27029438 PMC4976226

[B107] Todd N. Josephs O. Zeidman P. Flandin G. Moeller S. Weiskopf N. (2017). Functional sensitivity of 2D simultaneous multi-slice echo-planar imaging: effects of acceleration on g-factor and physiological noise. *Front. Neurosci.* 11:158. 10.3389/fnins.2017.00158 28424572 PMC5372803

[B108] Tong Y. Frederick B. D. (2010). Time lag dependent multimodal processing of concurrent fMRI and near-infrared spectroscopy (NIRS) data suggests a global circulatory origin for low-frequency oscillation signals in human brain. *Neuroimage* 53 553–564. 10.1016/j.neuroimage.2010.06.049 20600975 PMC3133965

[B109] Tong Y. Frederick B. D. (2014). Tracking cerebral blood flow in BOLD fMRI using recursively generated regressors. *Hum. Brain Mapp.* 35 5471–5485. 10.1002/hbm.22564 24954380 PMC4206590

[B110] Triantafyllou C. Hoge R. D. Krueger G. Wiggins C. J. Potthast A. Wiggins G. C. (2005). Comparison of physiological noise at 1.5 T, 3 T and 7 T and optimization of fMRI acquisition parameters. *Neuroimage* 26 243–250. 10.1016/j.neuroimage.2005.01.007 15862224

[B111] Tsvetanov K. A. Henson R. N. A. Jones P. S. Mutsaerts H. Fuhrmann D. Tyler L. K. (2020). The effects of age on resting-state BOLD signal variability is explained by cardiovascular and cerebrovascular factors. *Psychophysiology* 2020:e13714. 10.1111/psyp.13714 PMC8244027 33210312

[B112] Tsvetanov K. A. Henson R. N. A. Tyler L. K. Davis S. W. Shafto M. A. Taylor J. R. (2015). The effect of ageing on fMRI: Correction for the confounding effects of vascular reactivity evaluated by joint fMRI and MEG in 335 adults. *Hum. Brain Mapp.* 36 2248–2269. 10.1002/hbm.22768 25727740 PMC4730557

[B113] Uludağ K. Dubowitz D. J. Yoder E. J. Restom K. Liu T. T. Buxton R. B. (2004). Coupling of cerebral blood flow and oxygen consumption during physiological activation and deactivation measured with fMRI. *Neuroimage* 23 148–155. 10.1016/j.neuroimage.2004.05.013 15325361

[B114] Van Dijk K. R. A. Hedden T. Venkataraman A. Evans K. C. Lazar S. W. Buckner R. L. (2010). Intrinsic functional connectivity as a tool for human connectomics: theory, properties, and optimization. *J. Neurophysiol.* 103 297–321. 10.1152/jn.00783.2009 19889849 PMC2807224

[B115] Vogt K. M. Ibinson J. W. Schmalbrock P. Small R. H. (2011). Comparison between end-tidal CO2 and respiration volume per time for detecting BOLD signal fluctuations during paced hyperventilation. *Magn. Reson. Imaging* 29 1186–1194. 10.1016/j.mri.2011.07.011 21908130 PMC3199369

[B116] Wang Q. Pelligrino D. A. Koenig H. M. Albrecht R. F. (1994). The role of endothelium and nitric oxide in rat pial arteriolar dilatory responses to CO2 in vivo. *J. Cereb. Blood Flow Metab.* 14 944–951. 10.1038/jcbfm.1994.126 7929657

[B117] Wise R. G. Harris A. D. Stone A. J. Murphy K. (2013). Measurement of OEF and absolute CMRO2: MRI-based methods using interleaved and combined hypercapnia and hyperoxia. *Neuroimage* 83 135–147. 10.1016/j.neuroimage.2013.06.008 23769703 PMC4151288

[B118] Xu F. Uh J. Brier M. R. Hart J. Yezhuvath U. S. Gu H. (2011). The influence of carbon dioxide on brain activity and metabolism in conscious humans. *J. Cerebral Blood Flow Metab.* 31 58–67. 10.1038/jcbfm.2010.153 20842164 PMC3049465

[B119] Yao J. F. Yang H.-C. S. Wang J. H. Liang Z. Talavage T. M. Tamer G. G. Jr. (2021). A novel method of quantifying hemodynamic delays to improve hemodynamic response, and CVR estimates in CO2 challenge fMRI. *J. Cereb. Blood Flow Metab.* 2021:271678X20978582. 10.1177/0271678X20978582 PMC8327112 33444087

[B120] Zou Q. H. Zhu C. Z. Yang Y. Zuo X. N. Long X. Y. Cao Q. J. (2008). An improved approach to detection of amplitude of low-frequency fluctuation (ALFF) for resting-state fMRI: fractional ALFF. *J. Neurosci. Methods* 172 137–141. 10.1016/j.jneumeth.2008.04.012 18501969 PMC3902859

